# Clinical Indications, Utilization, and Funding of Bariatric Surgery in Europe

**DOI:** 10.1007/s11695-014-1537-y

**Published:** 2014-12-21

**Authors:** Oleg Borisenko, Zeynep Colpan, Bruno Dillemans, Peter Funch-Jensen, Jan Hedenbro, Ahmed R. Ahmed

**Affiliations:** 1 Synergus AB, Svardvagen 19, 182 33 Danderyd, Sweden; 2 Department of General Surgery, St-Jan’s Hospital, Brugge, Belgium; 3 Aarhus University, Aleris-Hamlet Hospital, Aarhus, Denmark; 4 Aleris Obesity, Lund University, Lund, Sweden; 5 Imperial College London, London, UK

**Keywords:** Bariatric surgery, Clinical indication, Reimbursement, Utilization, Health care policy

## Abstract

**Purpose:**

The objective of this study was to evaluate the current utilization, the level of endorsement by professional societies, and health technology assessment bodies, as well as the reimbursement levels for bariatric surgery in European countries.

**Materials and Methods:**

We performed an analysis of the indications for bariatric surgery based on national clinical and commissioning guidelines, current utilization of surgery, characteristics of patients who underwent surgery, and reimbursement tariffs in Belgium, Denmark, England, France, Germany, Italy, and Sweden. Data were obtained from national patient registries, administrative databases, and published literature for the year 2012.

**Results:**

Despite clear consensus outlined in clinical guidelines, significant differences were found in the eligibility criteria for surgery. Patients with no significant comorbidities were deemed eligible if they had a body mass index (BMI) of 40 or 50 kg/m^2^ in Denmark. Irrespective of the country, patients with comorbidities were eligible if they had a BMI of 35 kg/m^2^. The highest utilization of bariatric surgery (number of surgeries per 1 M population) was observed in Belgium (928), Sweden (761), and France (571) while Italy (128), England (117), and Germany (72) had the lowest utilization. There was a strong negative correlation between utilization and average BMI level of the patient population (*r* = −.909, *p* = 0.005). The annual per capita spending on surgery differed significantly between countries, ranging from €0.54 in Germany to €4.33 in Belgium.

**Conclusions:**

There are significant variations in the clinical indications, utilization, and funding of bariatric surgery in European countries.

**Electronic supplementary material:**

The online version of this article (doi:10.1007/s11695-014-1537-y) contains supplementary material, which is available to authorized users.

## Introduction/Purpose

Bariatric surgery is highly effective for patients with severe obesity who fail to achieve sustained weight loss with conventional treatments. Although there is agreement regarding the clinical indications for surgery, little is known about the relative use of bariatric surgery in European countries. The objectives of this study were to evaluate and compare the current use of bariatric surgery, the degree of endorsement by professional societies, and health technology assessment bodies, as well as the level of reimbursement.

## Materials and Methods

We analyzed the indications for bariatric surgery, current use of surgery, characteristics of patients undergoing surgery, and reimbursement tariffs from seven European countries (Belgium, Denmark, England, France, Germany, Italy, and Sweden).

Information regarding the clinical indications was obtained from national clinical and commissioning guidelines for obesity and bariatric surgery. Bariatric surgical patients’ clinical characteristics were retrieved from patient registries (the Danish Bariatric Surgery Registry for Denmark, the Scandinavian Obesity Surgery Registry for Sweden, and the National Bariatric Surgery Registry for the United Kingdom), large national studies (France [1] and Germany [2]), and large single-center reports (Belgium [3] and Italy [4]).

Data on the annual number of adjustable gastric banding, gastric bypass, and sleeve gastrectomy surgeries were obtained from patient registries for Denmark (2012), Italy (2012), and Sweden (2012). Data for Belgium, France, Germany, and England were obtained from national administrative databases that included the Belgian Technical Unit for Analysis of Hospital Data (2010), the French Technical Agency for Hospitalization Information (2012), Hospital Episode Statistics for England (2011–2012), and the German Diagnostic-Related Group (DRG) data (2011). For England and Germany, a combination of procedure and diagnosis codes was used to estimate the number of surgical procedures performed (Table 1). For France, only the procedure codes were used. For Belgium, the number of patient cases in the All Patient Reported DRG system for 2010 was used as a proxy for the number of surgical procedures. Data about number of procedures were obtained only for residents of the country, where possible. The rates of all surgical procedures per 1 million (M) population were calculated using population size data from the Organization for Economic Cooperation and Development [5]. The prevalence of obesity in the adult population was obtained from the World Health Organization [6].

**Table 1:** Procedure and disease codes used for the estimation of bariatric surgery volume

Country	Procedure codes	Diagnosis codes	DRG codes
Belgium	–	–	APR DRG 403
France	HFFA011, HFFC018, HFCC003, HFCA001, HFMA009, HFMC007, HFKA002, HFKC001	–	–
Germany	5–434.50, 5–434.51, 5–434.52, 5–436.11, 5–445.01, 5–445.02, 5–445.10, 5–445.11, 5–445.12, 5–445.20, 5–445.21, 5–445.22, 5–445.40, 5–445.41, 5–445.42, 5–445.50, 5–445.51, 5–445.52, 5–445.×1, 5–445.*x*2, 5–445.×3, 5–448.b0, 5–448.b1, 5–448.b2, 5–448.bx, 5–448.c0, 5–448.c1, 5–448.c2, 5–448.c3, 5–448.cx	E66, E66.0, E66.1, E66.2, E66.8, E66.9	–
England	G28.1, G28.2, G28.3, G28.4, G28.5, G28.8, G28.9, G30.1, G30.2, G30.3, G31.0, G31.1, G31.2, G31.4, G31.6, G31.8, G31.9, G32.0, G32.1, G32.3, G32.4, G32.5, G32.8, G32.9, G33.0, G33.1, G33.3, G33.5, G33.6, G33.8, G33.9, G5.11	E66	–

Pearson’s product-moment correlation, Spearman’s rank-order correlation, and linear regression were performed to assess the relationship between the number of surgeries performed annually (per 1 M population) and the average BMI, the prevalence of comorbidities in patients undergoing surgery, and the obesity prevalence.

Interrupted time series analysis using autoregressive integrated moving average and time series regression techniques were performed to evaluate the impact of the new 2010 guidelines for the use of bariatric surgery in Denmark [7]. An estimate of the number of surgeries performed in 2013 was obtained from experts in the field. But because of the limited number of observations available between 2007 and 2013, autoregression was not possible. Statistical analysis was performed using SPSS version 20 (IBM Corp., Armonk, New York, USA).

Reimbursement tariffs for each country were obtained through their respective national DRG by combining the relevant procedure and diagnosis codes in the grouping software (for Denmark, France, Germany, Sweden, and England) [8–12]. For Italy, relevant DRG code was found in national clinical guidelines [13]. For Belgium, the value for the relevant tariff in the All Patient Refined DRG Project was available only for 2010. The total annual spending on bariatric surgery was estimated by multiplying the relevant averaged national public tariff and the number of surgeries performed. In France, separate tariffs for public and private providers were applied. A weighted average was obtained for the tariff for each surgical technique, and all cost data are presented in 2012 Euros.

## Results

### Clinical Indications

Table 2 shows a summary of the clinical indications for bariatric surgery. With the exception of Denmark and England, the entry BMI level for surgery for patients with no serious comorbidities is 40 kg/m^2^. In Denmark, clinical guidelines recommend surgery at a BMI of 40 kg/m^2^, while commissioning guidelines determine reimbursement only for patients with a BMI of ≥50 kg/m^2^. In England, the entry BMI level for surgery is 40 kg/m^2^ with requirements for an intensive weight loss program for at least 12–24 months (6 months for those with BMI > 50 kg/m^2^). For patients with comorbidities, the minimum BMI was 35 kg/m^2^ for all countries, although in England, this group of patients must still undertake an intensive weight loss program for 12–24 months. The most common comorbidities include type 2 diabetes (all countries), hypertension (all countries except Sweden), knee osteoarthritis (Denmark, France, Italy, Sweden, and England), and obstructive sleep apnoea (Belgium, Denmark, France, and Sweden).

**Table 2:** National guidance for bariatric surgery

Country	BMI level, kg/m^2^		Comorbidities	Requirements for conservative treatment prior to bariatric surgery	Source
	Without comorbidities	With comorbidities			
Belgium	40	35	T2DM, hypertension, OSA	Not available	[14]
Denmark	40	35	T2DM, hypertension, OSA, PCOS, knee osteoarthritis	Sustainable weight loss not achieved by conventional treatment	[15]
	50	35			[16]
England	40	35	T2DM, hypertension, OSA, knee osteoarthritis	Failure of non-surgical methods for at least 6 months	[17]
	50			Bariatric surgery is recommended as a first-line treatment option	
	40			Weight loss programme for 12–24 months	[18]
	50			Weight loss programme for 6 months minimum	
France	40	35	T2DM, hypertension, OSA, knee osteoarthritis	Failure of nutritional, dietary and medical treatment; psychotherapy conducted for 6–12 months; absence of sufficient weight loss or lack of maintenance of weight loss	[19]
Germany	40	35	T2DM, hypertension	Failure of conservative management	[20]
Italy	40	35	T2DM, hypertension, knee osteoarthritis, severe psychological problems	Failure of proper medical treatment including inadequate weight loss or poor maintenance of weight loss	[13]
Sweden	40	35	T2DM, OSA, pregnancy issues, knee osteoarthritis	Failure of weight loss using non-surgical methods	[21]

*BMI* body mass index, *OSA* obstructive sleep apnea, *PCOS* polycystic ovary syndrome, *T2DM* type 2 diabetes

### Patient Characteristics

A comparison of the clinical indications and patient characteristics for bariatric surgery is shown in Table 3.

**Table 3:** Comparison of clinical indications and characteristics of the patient population who underwent bariatric surgery

Country	Cohort	BMI level with no comorbidities	BMI level with at least comorbidity	Prevalence of comorbidities			
				T2DM	Hypertension	OSA	Knee osteoarthritis
Belgium	Indication	40	35	Yes	Yes	Yes	No
	Real cohort	38		9 %	28 %	3 %	NA
Denmark	Indication	40/50	35	Yes	Yes	Yes	Yes
	Real cohort	45		23 %	32 %	11 %	NA
England	Indication	40/50	35	Yes	Yes	Yes	Yes
	Real cohort	50.6		24 %	32 %	16 %	53 %
France	Indication	40	35	Yes	Yes	Yes	Yes
	Real cohort	43.7		10 %	22 %	12 %	NA
Germany	Indication	40	35	Yes	Yes	No	No
	Real cohort	48.8		20 %	57 %	21 %	44 %
Italy	Indication	40	35	Yes	Yes	No	Yes
	Real cohort	46.2		NA	NA	NA	NA
Sweden	Indication	40	35	Yes	No	Yes	Yes
	Real cohort	42.8		18 %	43 %	9 %	NA

*BMI* body mass index, *NA* not available, *OSA* obstructive sleep apnea, *T2DM* type 2 diabetes

**Fig. 1:**
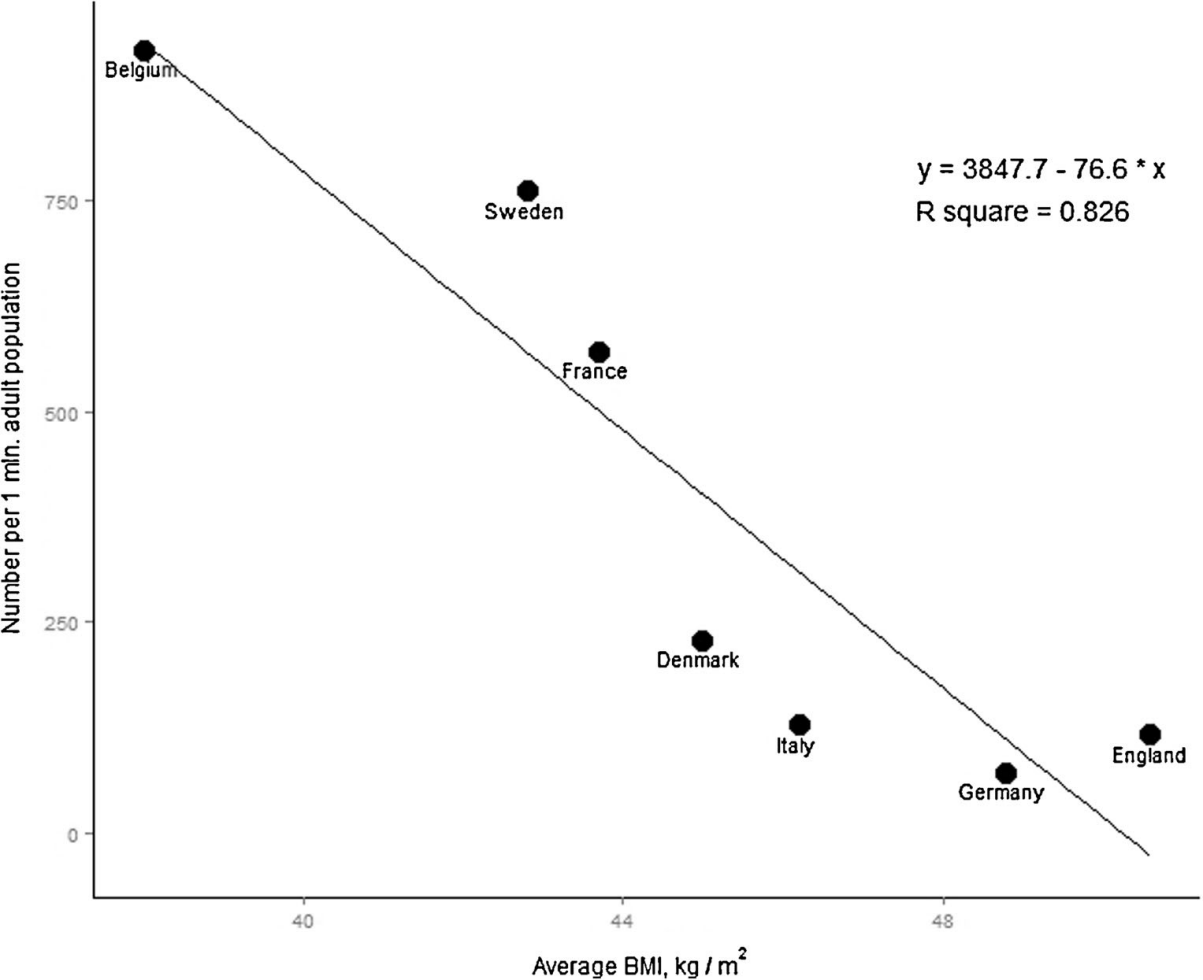
Linear regression for the annual number of surgeries per 1 M population and the average BMI level of the patient population who underwent surgery

**Fig. 2:**
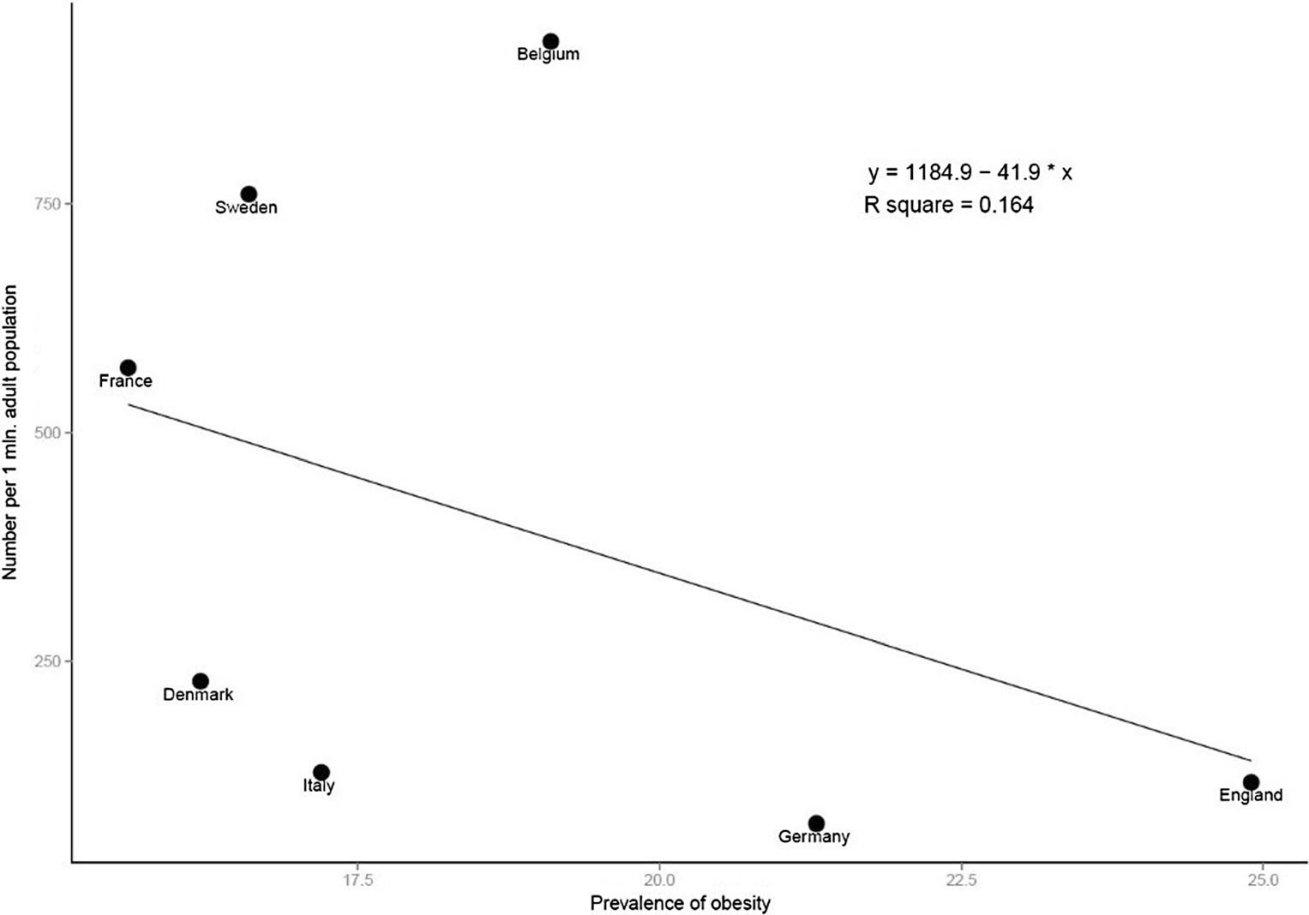
Linear regression for the annual number of surgeries per 1 M population and the prevalence of obesity (percentage of population) in each country

There was a strong negative correlation between the number of surgeries performed annually (per 1 M population) and the average patient BMI (*r* = −.909, *p* = 0.005). Figure 1 shows the linear regression analysis. There was a moderate, but not statistically significant correlation found between the number of surgeries performed annually (per 1 M population) and the prevalence of obesity (*r* = −.404, *p* = 0.368) (Fig. 2 shows the linear regression analysis). There was a strong negative but not statistically significant correlation between the number of surgeries performed annually per 1 M population and the prevalence of type 2 diabetes (*r* = −.793, *p* = 0.060), a moderate negative but not statistically significant correlation with the prevalence of hypertension (*r*_*s*_ = −.522, *p* = 0.228), and a significant strong negative correlation with the prevalence of obstructive sleep apnea (*r* = −.887, *p* = 0.019).

### Use and Funding of Bariatric Surgery

**Fig. 3:**
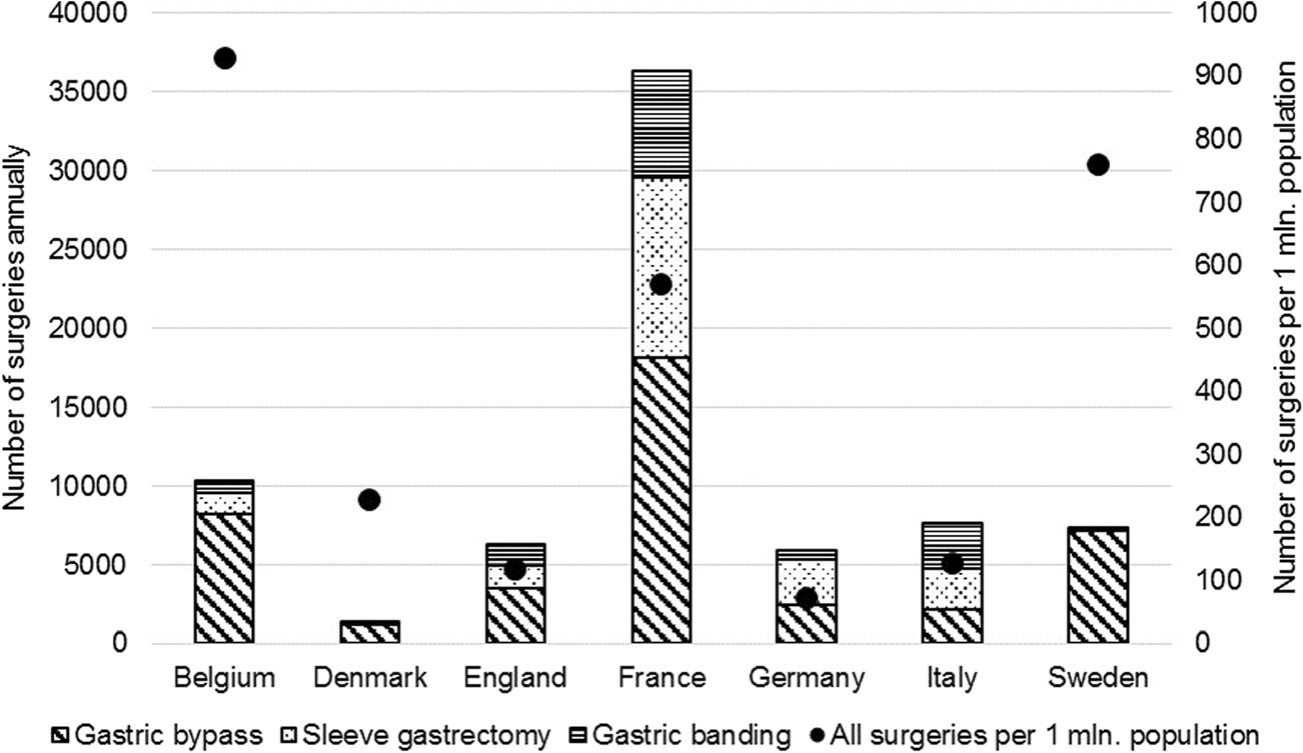
Number of bariatric surgeries performed in European countries. Note: types of surgery are plotted against the *left vertical axis. Circles* represent number of surgeries per 1 million of the population in each country and are plotted against the *right vertical axis*

The use of the three most common bariatric surgeries in 2012 is shown in Fig. 3. Gastric bypass is the most dominant procedure in all countries with the highest use in Sweden (98 %), Denmark (96 %), and Belgium (80 %). Gastric banding is used significantly only in France (19 %), England (21 %), and Italy (37 %). Maximum surgical rates (per 1 M population) are found in Belgium (928), Sweden (761), and France (571), while the lowest rates are in England (117) and Germany (72).

Estimates of annual spending on bariatric surgery in 2012 are shown in Table 4. Per capita spending on bariatric surgery differs more than eight times between the countries with the lowest and the highest spending, ranging from €0.54 in Germany to €4.33 in Belgium.

**Table 4:** Spending on bariatric surgery

Country	Type of surgery	Weighted average reimbursement tariffs, €	DRG group	Specificity of DRG in relation to different types of surgery	Specificity of DRG in relation to complications	Total spending on bariatric surgery, € million	Total per capita spending, €
Belgium	All types	4668	APR-DRG 403	Non-specific	Non-specific	48	4.33
Denmark	GBP	4327	DRG 1004	Non-specific	Non-specific	5	0.98
	SG	3742	DRG 2601				
	GB	4327	DRG 1004				
England	GBP	5999	FZ04A, FZ04B	Specific	Specific (2 levels of severity)	33	0.62
	SG						
	GB	2792	FZ05A, FZ05B		Specific (2 levels of severity)		
France	GBP	7314	10C13	Specific	Specific (4 levels of severity)	243	3.77
	SG	7314	10C13				
	GB	3524	10C09 + Nomenclature tariff for band				
Germany	GBP	7667	K04A	Specific	Non-specific	44	0.54
	SG	7667	K04A				
	GB	5725	K04B				
Italy	All types	5681	DRG 288	Non-specific	Non-specific	43	0.73
Sweden	GBP	5012	L08E, L08C, L08A	Non-specific	Specific (3 levels of severity)	36	3.81
	SG						
	GB						
	SG						
	GB	2792	FZ05A, FZ05B		Specific (2 levels of severity)		

*GB* gastric banding, *GBP* gastric bypass, *SG* sleeve gastrectomy

### Summary of Appraisal, Commissioning, and Quality Assurance for Bariatric Surgery in European Countries

#### Belgium

There are no national clinical guidelines for bariatric surgery in Belgium. In 2006, the Belgian Health Care Knowledge Centre issued a report that confirmed the effectiveness and cost-effectiveness of surgery versus nonsurgical treatment [14]. There is no national quality registry for bariatric surgery currently in place in Belgium, and since the Health Care Knowledge Centre report was published, no other national reviews on the status of bariatric surgery have been presented.

#### Denmark

In 2007, the Danish Centre for Health Technology Assessment issued a report on bariatric surgery that supported the use of gastric bypass and gastric banding [22]. Recommendations for surgery provision were issued in 2008 under the supervision of the National Board of Health [15]. In 2010, based on a review of the use of bariatric surgery in Denmark, the Danish Regions issued new, stricter guidelines for bariatric surgery [16]. The rationale included the overuse of surgery in patients with fewer comorbidities, the rapid increase in the number of surgeries, and the high complication rate. The goal of the treatment guidelines was to reduce the number of surgeries by 40 % to provide resources for treatment in other areas.

**Fig. 4:**
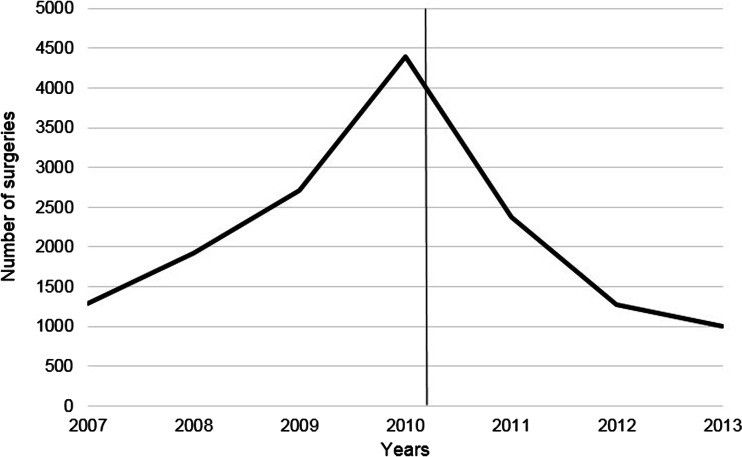
Number of bariatric surgeries performed in Denmark between 2007 and 2013. Note: *the black line* indicates when the clinical indications were changed (2010, effective from 2011)

Interrupted time series analysis showed that the number of bariatric surgeries decreased significantly in 2010 following changes to the clinical indications (Fig. 4). The guidelines reduced the number of procedures by 2879 after 1 year (standard error 566, 95 % confidence interval 444–5314 cases). Details of the analysis are provided in the Supplemental Material.

Between 2010 and 2012, although the prevalence of comorbidities increased slightly (5 % for type 2 diabetes, 4 % for hypertension, and 3 % for obstructive sleep apnoea) [23–25], the average patient BMI remained the same at 45 kg/m^2^, but the median decreased to 44 kg/m^2^. A high-quality registry for bariatric surgery has been implemented at the Danish national level since 2010, which annually monitors performance using eight indicators [26].

#### France

The national health technology assessment body, the French National Authority for Health, issued recommendations for bariatric surgery in 2009 [19]. In 2007, national bariatric surgery was established under the umbrella of the French and French-speaking Society of Obesity Surgery, although general reports from the registry are not available. In general, the clinical and policy environment is favorable toward bariatric surgery.

#### Germany

The value of bariatric surgery in Germany was evaluated in 2008 by the German Institute of Medical Information and Documentation [27]. Despite acknowledgement of the short- and mid-term effectiveness as well as the cost-effectiveness of bariatric surgery, the evaluators recommended restricted reimbursement. Currently, there is an ongoing national quality assurance study on the safety and efficacy of bariatric surgery although follow-up is limited to 1 year [2]. The most recent German clinical guidelines were issued in 2009. In general, bariatric surgery is well accepted by the medical community, but a bottleneck in patient care is created by the insurance companies, which require extensive and not well-defined pre-surgical conservative treatments.

#### Italy

In 2008, the Italian Society of Obesity and Metabolic Diseases issued clinical guidelines and a state-of-the-art report [13]. Regional inequalities accessing surgery and an unbalanced reimbursement tariff were among the barriers for broader implementation of bariatric surgery. Reimbursement tariffs for bariatric surgery also differed significantly between regions. The Italian Society of Obesity and Metabolic Diseases maintains a national registry of bariatric surgery [28].

#### Sweden

In 2002, the Swedish Council on Health Technology Assessment produced a report on the treatment of obesity and concluded that “in people with severe obesity, surgical treatment has positive, well-documented long-term effects on weight, quality of life, and morbidity from diabetes” [29]. In 2008, a mini-health technology appraisal was performed in one of the Swedish regions (Region Västra Götaland), which concluded that there was good evidence for weight loss effect and reasonable safety compared with other procedures, but limited evidence for the impact on mortality and diabetes [30]. The latest clinical indications for bariatric surgery were established in 2011 [21]. Sweden has hosted a comprehensive, high-quality Scandinavian Obesity Surgery Registry since 1998 [31] that includes routine 2-year follow-up following the surgical procedure. Despite a favorable environment, the number of cases has decreased slightly in 2013 compared with 2011–2012.

#### United Kingdom/England

In the UK, the economic value of bariatric surgery was evaluated in a number of studies: a health technology assessment report in 2008 [32], an economic report by the Office of Health Economics [33], and a short appraisal in Scotland in 2012 [34]. Bariatric surgery was shown to be effective, reasonably safe, and cost-effective (cost saving analysis from a societal perspective) in UK settings. The National Institute of Clinical and Care Excellence issued the latest clinical guidelines on obesity in 2006, which endorsed bariatric surgery. On 1 April 2013, the National Health Services in England took over commissioning of bariatric surgery from the primary care setting and added a supplementary requirement for all patients to undergo a 12–24-month (6 months for BMI ≥ 50 kg/m^2^) “Tier 3” lifestyle intervention program in the community before being referred for bariatric surgery [18]. A review by the English Health and Social Care Information Centre showed that the number of bariatric surgeries decreased by 8.8 % from 2012 to 2013 compared with 2011–2012 [35]. A further downturn in bariatric surgery is expected for 2013–2014 as prospective patients get diverted to Tier 3 programs before surgery.

## Conclusions

This study provides an overview of the indications, use, and funding of bariatric surgery in patients from seven European countries. Our analysis has highlighted several significant differences between countries.

The indications for bariatric surgery differ significantly among countries despite strong agreement on the clinical guidelines. Interestingly, with increase of utilization of surgery BMI level is lowering, which illustrates inclusion of broader group of patients, although in all countries, indication remains within recommended limit. Despite the availability of substantial evidence for the safety and efficacy of bariatric surgery, reimbursement recommendations in Denmark for the coverage of surgery were changed, raising the BMI entry level to 50 kg/m^2^, while England has insisted on mandatory preoperative weight loss programs for which no supporting evidence exists. These changes are not consistent with the current evidence demonstrating the clinical benefit of bariatric surgery in patients with morbid obesity. If left untreated, these patients have significant unmet needs and careful consideration of the clinical, and economic impact of policy changes is required.

The Danish example particularly demonstrates, on one hand, the example of data-driven decision-making in bariatric surgery, but on the other hand, the lack of comprehensive and rational evidence-based disinvestment policy in health care in general across different disease areas. This means that as health care systems operate under substantial resource constraints, for sustainability of a system, it is required to consider both investment (reimbursement of new treatment methods) and disinvestment (termination of reimbursement of noneffective or noncost-effective methods). Tightening reimbursement criteria for bariatric surgery is some form of disinvestment, which, first, should be based on solid scientific ground and, second, should be based on comparison of benefits and costs of bariatric surgery with health care intervention in other clinical areas. In different European countries, bariatric surgery was found to be either very cost-effective or cost-saving [32, 36–40] and, in general, more cost-effective compared with many technologies in health care. This was supported by an analysis of potential efficiency gains that used cost-effectiveness evidence to reallocate Medicare expenditures in the USA where bariatric surgery was considered worth prioritization in comparison with many other services [41].

In our analysis, the highest use of bariatric surgery was observed in Belgium, Sweden, and France. As a total spending per capita, these three countries also had the highest level of spending on bariatric surgery. Moreover, high utilization of surgery is not explained by medical tourism, for example, in Belgium, which was not included into analysis. Nevertheless, in all countries, even with a high level of surgery use, the provision of care is still far below demand. Indeed, a recent English study estimated that more than two million people were eligible for surgery according to the National Institute for Care and Clinical Excellence indications, but the actual annual number of surgeries was <10,000 cases [42]. What is the optimal use of bariatric surgery in the current reimbursement and clinical environment? Based on our comparative analysis of seven European countries, the Swedish model may be the ideal. It includes clinical indications based on a comprehensive health technology assessment process, the specificity of reimbursement tariffs, regular tariff updates, a tight and comprehensive quality assurance program that prevents the overuse and reduction of the quality of surgery, the lack of additional barriers from service commissioners, the understanding of the relative value of surgery and conservative methods by the medical community, and a correlation between the clinical indications and patient characteristics.

There are several limitations to our study. We considered only three of the most common surgical techniques, and the inclusion of other surgeries might have changed the numbers for surgery use. We did not perform a comparison of outcomes of bariatric surgery in different countries as the efficacy and outcomes might depend highly on patient case mix, the number of hospitals involved, and other factors.

There are significant variations in the clinical indications, use, and funding of bariatric surgery in European countries. The update and revision of current clinical recommendations, based on the latest evidence available, are required to ensure optimal access to effective treatment options.

## Electronic supplementary material

Below is the link to the electronic supplementary material.

ESM 1 (DOCX 20 kb)
