# Supplementary material for: Clinical Indications, Utilization, and Funding of Bariatric Surgery in Europe
Source: Obes Surg. 2014 Dec 21;25(8):1408–16. doi: 10.1007/s11695-014-1537-y (PMC4498278; doi:10.1007/s11695-014-1537-y)
Supplement: Supplementary file 1 — (DOCX 20 kb) [file 11695_2014_1537_MOESM1_ESM.docx]

**Supplemental material for manuscript**

**“Clinical Indications, Utilization and Funding of Bariatric Surgery in Europe”**

**Interrupted time series analysis**

Data about annual utilization of bariatric surgery in Denmark between 2007 and 2013 used for analysis are presented in Table.

**Table. Number of bariatric surgeries, performed annually in Denmark between 2007 and 2013**

| Year | Number of surgeries |
| --- | --- |
| 2007 | 1283 |
| 2008 | 1921 |
| 2009 | 2711 |
| 2010 | 4397 |
| 2011 | 2368 |
| 2012 | 1276 |
| 2013 | 1000 |

**Estimation of difference between slopes (before and after 2011)**

| **Model Fit** | | | | | | | | | | | |
| --- | --- | --- | --- | --- | --- | --- | --- | --- | --- | --- | --- |
| Fit Statistic | Mean | SE | Minimum | Maximum | Percentile | | | | | | |
|  |  |  |  |  | 5 | 10 | 25 | 50 | 75 | 90 | 95 |
| Stationary R-squared | ,950 | . | ,950 | ,950 | ,950 | ,950 | ,950 | ,950 | ,950 | ,950 | ,950 |
| R-squared | ,950 | . | ,950 | ,950 | ,950 | ,950 | ,950 | ,950 | ,950 | ,950 | ,950 |
| RMSE | 371,137 | . | 371,137 | 371,137 | 371,137 | 371,137 | 371,137 | 371,137 | 371,137 | 371,137 | 371,137 |
| MAPE | 12,371 | . | 12,371 | 12,371 | 12,371 | 12,371 | 12,371 | 12,371 | 12,371 | 12,371 | 12,371 |
| MaxAPE | 21,317 | . | 21,317 | 21,317 | 21,317 | 21,317 | 21,317 | 21,317 | 21,317 | 21,317 | 21,317 |
| MAE | 227,429 | . | 227,429 | 227,429 | 227,429 | 227,429 | 227,429 | 227,429 | 227,429 | 227,429 | 227,429 |
| MaxAE | 373,600 | . | 373,600 | 373,600 | 373,600 | 373,600 | 373,600 | 373,600 | 373,600 | 373,600 | 373,600 |
| Normalized BIC | 12,945 | . | 12,945 | 12,945 | 12,945 | 12,945 | 12,945 | 12,945 | 12,945 | 12,945 | 12,945 |

| **Model Statistics** | | | | | | |
| --- | --- | --- | --- | --- | --- | --- |
| Model | Number of Predictors | Model Fit statistics | Ljung-Box Q(18) | | | Number of Outliers |
|  |  | Stationary R-squared | Statistics | DF | Sig. |  |
| Outcomes-Model_1 | 3 | ,950 | . | 0 | . | 0 |

| **ARIMA Model Parameters** | | | | | | | | |
| --- | --- | --- | --- | --- | --- | --- | --- | --- |
|  | | | | | Estimate | SE | t | Sig. |
| Outcomes-Model_1 | Outcomes | No Transformation | Constant | | 45,000 | 454,549 | ,099 | ,927 |
|  | Time_period | No Transformation | Numerator | Lag 0 | 1013,200 | 165,978 | 6,104 | ,009 |
|  | Phase | No Transformation | Numerator | Lag 0 | 5607,000 | 1652,847 | 3,392 | ,043 |
|  | Interact | No Transformation | Numerator | Lag 0 | -1697,200 | 310,516 | -5,466 | ,012 |

**Estimation of effect at 1 year after change of clinical indication for bariatric surgery**

| **Model Fit** | | | | | | | | | | | | |
| --- | --- | --- | --- | --- | --- | --- | --- | --- | --- | --- | --- | --- |
| Fit Statistic | Mean | SE | Minimum | Maximum | Percentile | | | | | | | |
|  |  |  |  |  | 5 | 10 | 25 | 50 | 75 | 90 | 95 |  |
| Stationary R-squared | ,950 | . | ,950 | ,950 | ,950 | ,950 | ,950 | ,950 | ,950 | ,950 | ,950 |  |
| R-squared | ,950 | . | ,950 | ,950 | ,950 | ,950 | ,950 | ,950 | ,950 | ,950 | ,950 |  |
| RMSE | 371,137 | . | 371,137 | 371,137 | 371,137 | 371,137 | 371,137 | 371,137 | 371,137 | 371,137 | 371,137 |  |
| MAPE | 12,371 | . | 12,371 | 12,371 | 12,371 | 12,371 | 12,371 | 12,371 | 12,371 | 12,371 | 12,371 |  |
| MaxAPE | 21,317 | . | 21,317 | 21,317 | 21,317 | 21,317 | 21,317 | 21,317 | 21,317 | 21,317 | 21,317 |  |
| MAE | 227,429 | . | 227,429 | 227,429 | 227,429 | 227,429 | 227,429 | 227,429 | 227,429 | 227,429 | 227,429 |  |
| MaxAE | 373,600 | . | 373,600 | 373,600 | 373,600 | 373,600 | 373,600 | 373,600 | 373,600 | 373,600 | 373,600 |  |
| Normalized BIC | 12,945 | . | 12,945 | 12,945 | 12,945 | 12,945 | 12,945 | 12,945 | 12,945 | 12,945 | 12,945 |  |

| **Model Statistics** | | | | | | | | | | | | | | |  |
| --- | --- | --- | --- | --- | --- | --- | --- | --- | --- | --- | --- | --- | --- | --- | --- |
| Model | | Number of Predictors | | Model Fit statistics | | Ljung-Box Q(18) | | | | | | | Number of Outliers | |  |
|  |  |  |  | Stationary R-squared | | Statistics | | | DF | | Sig. | |  |  |  |
| Outcomes-Model_1 | | 3 | | ,950 | | . | | | 0 | | . | | 0 | |  |
| **ARIMA Model Parameters** | | | | | | | | | | | | | | | |
|  | | | | | | | | Estimate | | SE | | t | | Sig. | |
| Outcomes-Model_1 | Outcomes | | No Transformation | | Constant | | | 45,000 | | 454,549 | | ,099 | | ,927 | |
|  | Pre_1 | | No Transformation | | Numerator | | Lag 0 | 1013,200 | | 165,978 | | 6,104 | | ,009 | |
|  | Post_1 | | No Transformation | | Numerator | | Lag 0 | -684,000 | | 262,434 | | -2,606 | | ,080 | |
|  | Phase | | No Transformation | | Numerator | | Lag 0 | -2879,000 | | 566,922 | | -5,078 | | ,015 | |

**Estimation of 95% confidence interval for effect at 1 year after change of clinical indication for bariatric surgery**

For estimation of confidence interval the following formula was used (18):

*Estimate of effect +/- tinv (0.05; df) * SE*, where:

- tinv – Function in MS Excel,
- df – degree of freedom
- SE – standard error of effect
